# Cryo-Imaging and Software Platform for Analysis of Molecular MR Imaging of Micrometastases

**DOI:** 10.1155/2018/9780349

**Published:** 2018-04-01

**Authors:** Mohammed Q. Qutaish, Zhuxian Zhou, David Prabhu, Yiqiao Liu, Mallory R. Busso, Donna Izadnegahdar, Madhusudhana Gargesha, Hong Lu, Zheng-Rong Lu, David L. Wilson

**Affiliations:** ^1^Department of Biomedical Engineering, Case Western Reserve University, 10900 Euclid Avenue, Cleveland, OH 44106, USA; ^2^BioInVision Inc., Suite E, 781 Beta Drive, Cleveland, OH 44143, USA; ^3^Department of Radiology, Case Western Reserve University, 10900 Euclid Avenue, Cleveland, OH 44106, USA

## Abstract

We created and evaluated a preclinical, multimodality imaging, and software platform to assess molecular imaging of small metastases. This included experimental methods (e.g., GFP-labeled tumor and high resolution multispectral cryo-imaging), nonrigid image registration, and interactive visualization of imaging agent targeting. We describe technological details earlier applied to GFP-labeled metastatic tumor targeting by molecular MR (CREKA-Gd) and red fluorescent (CREKA-Cy5) imaging agents. Optimized nonrigid cryo-MRI registration enabled nonambiguous association of MR signals to GFP tumors. Interactive visualization of out-of-RAM volumetric image data allowed one to zoom to a GFP-labeled micrometastasis, determine its anatomical location from color cryo-images, and establish the presence/absence of targeted CREKA-Gd and CREKA-Cy5. In a mouse with >160 GFP-labeled tumors, we determined that in the MR images every tumor in the lung >0.3 mm^2^ had visible signal and that some metastases as small as 0.1 mm^2^ were also visible. More tumors were visible in CREKA-Cy5 than in CREKA-Gd MRI. Tape transfer method and nonrigid registration allowed accurate (<11 *μ*m error) registration of whole mouse histology to corresponding cryo-images. Histology showed inflammation and necrotic regions not labeled by imaging agents. This mouse-to-cells multiscale and multimodality platform should uniquely enable more informative and accurate studies of metastatic cancer imaging and therapy.

## 1. Introduction

Today, primary tumor masses are clinically controlled with surgical, drug, and radiation therapies, but the ability to control metastatic cancer is limited and over 90% of cancer patients die from metastases. Arguably, metastatic cancer is the most important front in the war on cancer, but it is hampered by an inability to see the enemy. Preclinical research on micrometastases (1 cell to about 2 mm), scattered throughout the body, is limited because histology is impractical and because traditional in vivo imaging has insufficient resolution and contrast for reliable detection. As a result, often targeting of imaging agents and theranostics is based on primary tumors with different genes, expression, and/or environment than scattered metastases. We are creating a preclinical, quantitative, and Cancer Imaging and Therapy Platform (CITP) which will allow one to study cancer biology and optimize pipelines of technologies (e.g., imaging agents, imaging methods, targeted nanotherapeutics, and tumor models), especially for metastatic and invasive cancers.

In this report, we describe the CITP technology and, as a demonstration, apply it to molecular MR imaging. This builds upon a previous application report where we used much manual analysis [1]. The platform includes experimental methods, small animal imaging, cryo-imaging, histology, and software “glue.” The central technology is cryo-imaging, a section-and-image ex vivo imaging technique which provides anatomical color and molecular fluorescence, single cell sensitivity, and 3D microscopic imaging over vast volumes, as large as an entire mouse [1–4]. With the use of fluorescent-protein-labeled tumors, cryo-imaging provides the gold standard for identification of metastatic tumors. The CITP concept is best understood by an example of CREKA-Gd and CREKA-Cy5 imaging agents targeting breast cancer metastases [1]. We injected CREKA-Gd and CREKA-Cy5 agents visible in MR and red fluorescence, respectively, into a mouse with GFP-labeled metastases. High resolution MR imaging was followed by cryo-imaging. Selected histology sections were obtained using a tape system that ensured spatial fidelity. Following registration, we had a green fluorescence GFP tumor volume, a high resolution color anatomy volume, red fluorescence and MR imaging agent volumes, and selected histology, in which we could identify tumors and imaging agent labeling. We identified micrometastases GFP tumors, determined the presence of red fluorescent imaging agent using highly sensitive cryo-fluorescence, determined if there was detectable MR signal, and examined histology for identification of tumor heterogeneity. In this way, we were able to identify that CREKA-Gd labeled an abundance of small and micrometastases.

Other methods exist to provide a metastasis gold standard, but they all have limitations as compared to cryo-imaging. In mouse models, an elegant approach for proving the location of a tumor is to use a sensitive reporter gene approach. Previous attempts to image whole mouse GFP expressing metastases were only efficient at shallow levels from the surface [5–8]. Fluorescence molecular tomography (FMT, Visen) tried to resolve this by using near infrared fluorescent labels which has better tissue penetration and less scattering and absorption. However, resolution and sensitivity of FMT are limited, and a limited number of fluorophores can be used because the system uses near infrared excitation lasers. Bioluminescence is quite sensitive, but the resolution is very poor due to light scatter, not allowing us to know if there is a single large tumor or many small ones. Two photon and confocal microscopy have been improved to image deeper sections with less photo bleaching by using near infrared lasers [9] but are still limited in the field of view. Although multiphoton intravital microscopy provides unique in vivo high resolution optical images that can be used to monitor tumor progression, invasion, and response to therapy [10], it is limited to small optical windows and cannot access all tissues in the mouse [11]. A common approach for proving a molecular imaging agent against disease is to obtain selected histological sections. However, it is typically quite difficult to prove spatial correspondence with an in vivo, whole mouse imaging modality. In addition, when sections are obtained, there is a potential for loss of the agent during histological processing unless the label is very tightly bound. Moreover, the histology approach necessarily creates a significant under sampling problem, making it unlikely to identify and evaluate false positive detection. It would not be easy to register any of these methods to exact locations for comparison to an in vivo imaging technology such as PET or MRI.

Our aim in this paper will be to describe the underlying experimental, software, imaging technologies, and their validation, that make CITP possible. As an example, we will demonstrate methods using molecular MR (CREKA-Gd) and fluorescence (CREKA-Cy5) imaging of metastases. CITP technologies include high resolution and sensitivity cryo-imaging, experimental treatments to aid registration, whole mouse nonrigid registration algorithms, interactive visualization/analysis software that can accommodate very large out-of-RAM image data sets, and a tape method and nonrigid registration to allow accurate registration of histology.

## 2. Experimental and Imaging Methods

### 2.1. Overview

**Figure 1:**
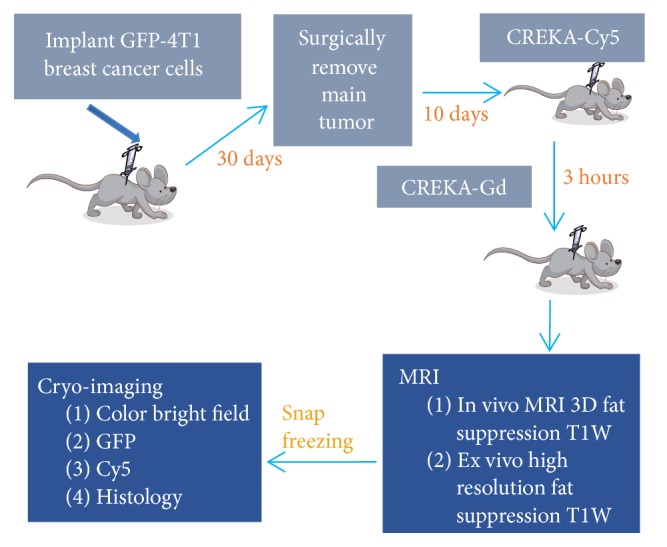
Experimental design for evaluation of CREKA imaging agents for a metastatic breast cancer model. GFP-labeled, 4T1 cells were injected in the fat pad. CREKA-Cy5 was injected in the tail vein for fluorescence evaluation. After 3 hours to allow clearance of nonbound agent, CREKA-Gd was injected. With MRI, we captured in vivo and high resolution ex vivo image volumes. After embedding in the freezing medium and snap freezing, we acquired microscopic, anatomical, colored episcopic and molecular, multispectral fluorescence cryo-image volumes. Optionally, selected histological sections were manually acquired using a tape system.

We developed a prototype software platform to support analyses similar to those done mostly manually in a previous application report [1]. Although this illustrates evaluation of imaging agents using the CREKA peptide, the methodology is equally valid for other cancer models, peptides, targets, and imaging modalities. Briefly, we created a spontaneous metastatic breast cancer, mouse model by orthotopic injection of 4T1-GFP-Luc2 cells in the fat pad. These cells can be imaged with bioluminescence and with multispectral fluorescence cryo-imaging. After 30 days, we surgically removed the main tumor mass and allowed metastases to grow for 10 more days. We injected CREKA-Cy5 in the tail vein and allowed 3 hours for tissue clearance. We followed this with tail vein injection of CREKA-Gd. In MR imaging we captured an* in vivo* scan using a 3D fat suppression sequence optimized for Gadolinium and another high resolution fat suppression sequence after animal sacrifice to be used in 3D registration. Mice were embedded in a freezing compound (O.C.T., Tissue-Tek®) and flash frozen using liquid nitrogen. In cryo-imaging, we acquired color episcopic, green fluorescence GFP tumor and red fluorescence CREKA-Cy5 image volumes. During cryo-imaging, we also acquired selected histological sections using a special adhesive film and performed H&E staining. The experimental design is illustrated in Figure 1. To recap, data include CREKA-Gd MRI, episcopic anatomy, GFP tumor, CREKA-Cy5, and histology. The platform enables registration, analysis, and visualization of these data to determine the efficiency of targeted molecular imaging of tumors. Experimental details of tumor model creation, CREKA injection, and animal handling can be found in our previous publication [1].

### 2.2. Imaging Details

Pre- and postinjection in vivo scans were performed using the following: fat suppression three-dimensional (3D) FLASH sequence with respiratory gating [repetition time (TR) = 25 ms, echo time (TE) = 2.8 ms, average = 3, 15° flip angle, in-plane field of view (FOV) = 6 cm, 18 mm slab thickness, resolution = 0.1172 × 0.09766 × 0.562 mm, and scan duration = 10 m 14 s]. The ex vivo high resolution MRI scan consisted of the following: fat suppression 3D FLASH, TR = 80 ms, TE = 2.8 ms, average = 3, 15° flip angle, in-plane FOV = 6 cm, 18 mm slab thickness, resolution = 0.1172 × 0.09766 × 0.1406 mm, and scan duration: 2 h 11 m.

In cryo-imaging, mice were imaged at 10.5 *μ*m × 10.5 *μ*m in-plane resolution and sectioned at 50 *μ*m using CryoViz™ (BioInVision, Inc, Cleveland Ohio). GFP and Cy5 images were acquired using a liquid crystal RGB filter, a dual band FITC/Cy5 fluorescence filter (Exciter: 51008x, Dichroic: 51008 bs, Emitter: 51008 m, Chroma, Rockingham, VT), and a low-noise monochrome camera. Typically, color reflection and green and red fluorescence volumes totaled over 120 GB for each mouse.

We acquired assorted histological sections from mice during cryo-imaging using CryoFilm™ [12]. Basically, a special adhesive tape is pressed against the block face of the sample. As it is sectioned, one collects the tape with the tissue attached. Then the tape with the tissue is stained and placed on a slide with a coverslip. We used a slide scanning system (Olympus VS120). Using the CryoFilm method, one can collect a whole mouse section. Because the section is attached to the tape, the geometry of the section is preserved. This ensures accurate registration of the histological image to the corresponding cryo-image. We performed HE staining. However, other types of IHC staining can be performed as well.

### 2.3. Measuring Volume Change Using CT

We designed a computed tomography (CT) experiment to estimate any volume change in a mouse due to freezing so as to help guide our image registration approach. In the experiment, we replicated conditions of the MRI-cryo-experiment. Following euthanization, we acquired a whole mouse CT volume after death. Then, we covered the animal with freezing medium and flash froze it in liquid nitrogen. We then acquired a second CT volume. Prior to the experiment, we scanned a phantom of water to make sure that the system was calibrated, giving a flat reading of zero HU (Hounsfield units). By comparing HU before and after freezing, we can compute density changes in tissues. CT numbers in Hounsfield units are given by

(1) $$\begin{array}{c} CT\left(\;x,y\;\right)=1000\frac{\mu \left(x,y\right)-\mu_{w}}{\mu_{w}}, \end{array}$$

where *μ*(*x*, *y*) is the linear attenuation coefficient and *μ*_*w*_ is a constant that represents the attenuation coefficient of water. We substituted *μ*(*x*, *y*) = (*μ*/*ρ*)*ρ*(*x*, *y*) where (*μ*/*ρ*) is the mass attenuation coefficient, a constant for a given material, and *ρ* = mass/volume. Since mass is unchanged with freezing, we get the expression below for the local ratio of volumes after and before freezing.

(2) $$\begin{array}{c} \frac{V_{after}}{V_{before}}=\frac{1000+CT_{before\;\;freezing}}{1000+CT_{after\;\;freezing}}. \end{array}$$

By taking CT numbers from the same region, one can easily compute the volume ratio over a local region in various organs such as the liver, kidneys, and brain.

## 3. Image Processing, Visualization, and Analysis

### 3.1. 3D Registrations of Cryo- and MRI Image Volumes

We used the episcopic cryo-image volume as the reference volume and registered all other image data (3D MRI and 2D histology) to it. Of course, the GFP and Cy5 image volumes were already perfectly registered to the episcopic data as they were acquired concurrently. We now describe the gray scale, rigid, affine, and nonrigid registration steps used to register the moving MRI volume to the reference 3D episcopic color volume.

There were important preprocessing steps. Color episcopic images were converted to gray scale using Gray = 0.2126 · R + 0.7152 · G + 0.0722 · B, where R, G, and B are the red, green, and blue channels, respectively. The associated parameters for each channel are chosen according to ITU-R Recommendation BT.709. We chose this method over some others as it weighs the green channel heavily, giving good contrast in cryo-images. The very high resolution gray scale episcopic image volume was downsampled to 0.1 × 0.1 × 0.1 mm using an antialiasing Lanczos filter [13]. This was done to make voxels isotropic and to bring the voxel size closer to the MRI voxel size, a step experimentally determined to be necessary for robust registration. High resolution MRI image volumes (typically 0.1172 × 0.0977 × 0.1406 mm) were used. We did not resample MRI prior to registration. Cryo- and MRI volumes were both linearly mapped to an 8-bit (0–255) grayscale range. For cryo-data, window/level was interactively used to adjust contrast and to obtain minimum and maximum values suitable for resampling data into 256-bin histogram. For MRI data with values typically ranging from 0 to 32,766, we linearly mapped 0–15,000 to an 8-bit range, as nearly all tissue voxels laid within this range. These mappings ensured that bins for the joint histogram in mutual information calculations were appropriately filled. Image volumes were cropped using rectangular cropping just outside the tissue to remove extra background. In Amira, we roughly registered the two volumes manually using rigid body translation prior to the start of automated registration. As long as there was good overlap and volumes were similarly oriented, automated registration was successful.

We employed a multiscale, sequential registration approach. At the coarsest scale, we used the algorithm described in [14, 15] for rigid and affine registration. Basically, this algorithm uses a block matching strategy where local displacements are estimated based on matching small blocks within the volume using the absolute value of the normalized cross correlation as a similarity measure. Although normalized cross correlation is not normally used for multimodality registration, the method uses those blocks having the highest variance and hence tends to match edges well. Essentially,* absolute* normalized cross correlation will match edges regardless of their polarity. A global estimate for a transformation is calculated from the multiple local displacements of blocks using trimmed least squares to provide a robust estimate. This block matching approach generated better transformations than other algorithms that use global mutual information to generate a rigid and affine transformation. Hence, we chose this algorithm to serve as initial registration for later refinement with nonrigid registration. There are multiple parameters that we optimized in this algorithm including block size, neighborhood search size, spacing/overlap between blocks, and rejection percentage for the trimmed least squares.

Nonrigid registration was applied using the well-established free form deformation (FFD) algorithm [16] which optimizes a lattice of points using bicubic spline between the points. A cost function (*C*_total_) is iteratively optimized using the gradient descent algorithm by varying displacements of the control points. Below, NMI is normalized mutual information; BE (bending energy) and JL (Jacobian) are regularization parameters; and *w*'s are empirically determined weights.

(3) $$\begin{array}{c} C_{total}=\left(\;1-w_{1}-w_{2}\;\right)NMI-\left(\;w_{1}\;\right)BE-\left(\;w_{2}\;\right)JL. \end{array}$$

Normalized mutual information (NMI) is used because of its ability to register multimodality images [17] and its improved characteristics with nonoverlapping tissues as compared to standard mutual information [18]. *H*(*I*_1_) and *H*(*I*_2_) are the marginal entropies of *I*_1_ and *I*_2_, and *H*(*I*_1_, *I*_2_) is the joint entropy.

(4) $$\begin{array}{c} NMI\left(\;I_{1},I_{2}\;\right)=\frac{H\left(I_{1}\right)+H\left(I_{2}\right)}{H\left(I_{1},I_{2}\right)}. \end{array}$$

The BE term is a smoothness constraint that penalizes any nonsmooth, unrealistic deformations [16, 19]. Basically, this penalty term is based on the second spatial derivative of the deformation field as shown below. Since the second derivative measures curvature, high values of BE indicate bending of the deformation field.

(5) $$\begin{array}{c} ΒΕ=\underset{x\in R^{3}}{\sum }\left[\;\overset{3}{\underset{i=1}{\sum }}{\left(\;\frac{\partial^{2}Τ\left(x\right)}{\partial {x_{i}}^{2}}\;\right)}^{2}+2\overset{}{\underset{\begin{array}{c} i,j\\ i\neq j \end{array}}{\sum }}{\left(\;\frac{\partial^{2}Τ\left(x\right)}{\partial x_{i}\partial x_{j}}\;\right)}^{2}\;\right]. \end{array}$$

The determinant of the Jacobian is used to measure any change in local “volume.” A value less or greater than one means expansion or compression, respectively. A penalty term is added which penalizes both compression and expansion equally [20, 21]. It is the logarithm of the absolute value of the determinant of the Jacobian (JL) as given below.

(6) $$\begin{array}{c} JL=\overset{}{\underset{x\in R^{3}}{\sum }}\log\left(\;\left|\;J_{T}\left(\;x\;\right)\;\right|\;\right), \end{array}$$

where

(7) JTx=det⁡∂T1x∂x1∂T1x∂x2∂T1x∂x3∂T2x∂x1∂T2x∂x2∂T2x∂x3∂T3x∂x1∂T3x∂x2∂T3x∂x3.

Although this was a multistep process, in the end, we saved spatial transformation equations and computed the registered floating volume in one step to limit the interpolation errors that would result with interpolations at each step. There are numerous registration parameters to optimize, including grid spacing, number of iterations, and number of levels for multiscale registration. In addition, registration quality depends on the weights, *w*_1_ and *w*_2_. In the next section, we describe registration experiments to achieve optimized values.

### 3.2. Finding Optimal 3D Registration Parameters

We performed experiments to optimize parameters for optimal registration. We optimized nonrigid registration regularization terms (*w*_1_ and *w*_2_) to give good quality registrations in the lung region, the most difficult region to register in our experiments. Other regions, except for the kidneys, were relatively insensitive to weight tuning. To optimize registration parameters, we performed an exhaustive grid search on *w*_1_ and *w*_2_. We registered the entire mouse, quantitatively evaluated registration in the lungs, and qualitatively assessed registration elsewhere. To quantitatively assess registration quality, we computed various metrics from segmented lungs. An expert, MQ with more than 5 years of experience in mouse imaging, manually segmented the lungs in the cryo-reference and moving MRI volume following rigid and affine registration. Segmentation was done once and used in all registration experiments. We created binary lung volumes from the segmentations and applied the transform from gray scale registration to the MRI binary volume. Pseudocode for the process is shown in Algorithm 1, with metrics given below.

Following registration of the binary volumes, we calculated three metrics. First, we measured the percent volume difference, VD [22], where |·| refers to a volume.

(8) $$\begin{array}{c} VD=2\frac{\left|Cryo\right|-\left|MRI\right|}{\left|Cryo\right|+\left|MRI\right|}. \end{array}$$

Second, we calculated the Dice coefficient to assess overlap between the two volumes [23]. Third, we measured the average 3D surface distance between the registered MRI lungs and the reference cryo-image lungs. Three-dimensional surfaces were converted to a set of points, and for each point on the first surface the minimum distance to a point on the second surface was determined. Results were averaged. Next, the process was repeated switching the role of the first and second surfaces. The distance in one direction is defined as follows [24]:

(9) $$\begin{array}{c} SD=\frac{1}{N}\overset{N}{\underset{n=1}{\sum }}\min\left(\;dist\left(\;Cryo_{boundary\left(n\right)},MRI_{boundary}\;\right)\;\right). \end{array}$$

### 3.3. Final Whole Mouse Evaluation of Registration Accuracy

Using optimized parameters, we visually and quantitatively evaluated the quality of registration throughout the mouse. Visually, we created checkerboard views in 3 orthogonal planes. This allows one to examine from one volume to the other, continuation of recognizable features such as the edges of organs and blood vessels. Some quantitative assessments were measure of lung registration quality as described above. In addition, we created a tool that enabled the selection of points of interest in the GFP and MRI volumes. The goal was to independently measure registration accuracy of metastases using the GFP fluorescence image. Note that this was not included in registration, which was done using the bright field volume. We computed 3D Euclidian distances between corresponding pairs of points in the GFP fluorescence and MRI volumes. For smaller metastases, we marked center points of corresponding tumors in GFP and MRI. For larger metastases, we employed shape irregularity to identify corresponding points at tumor edges and at tips of protrusions from the surface of tumors. For each mouse we evaluated several small and big metastases in different organs. Results were reported as mean and standard deviation of 3D Euclidian distances.

### 3.4. Acquisition of Histology Images and Their 2D Registration to 2D Cryo-Images

We selectively acquired large histological sections using a specialized adhesive film based method which preserved the geometry of the histological section. We typically used H&E staining. Images were scanned on a microscope-based slide scanner.

Histology images were registered to their corresponding 2D cryo-images. Since both episcopic color cryo-images and histology images contain rich features with many edges, we applied edge-based registration. We converted color images to grayscale and used grayscale normalized cross correlation on edge images as a similarity measure. To reduce the small cellular edge features in the histology images without correspondence in cryo-images, we performed iterative open/close morphological gray scale filtering. Following this step, edge strength images from histology images looked very similar to those from cryo-images, and normalized cross correlation was appropriate. We first performed 2D global registration with 3 degrees of freedom (translation, rotation, and scale) to transform the “moving” histology edge image to the “reference” bright field edge image. We used Nelder-Mead simplex to optimize the objective function. A B-spline based, free form, multiresolution algorithm was used to correct the minimal, remaining local distortion. The histology image was then transformed to match the cryo-image. The edge-based objective function led to much better results than mutual information. To evaluate registration quality visually, we used sector and edge displays. A quantitative assessment was done by manually measuring the Euclidian distance between matching points in cryo- and histology images.

### 3.5. Tumor Targeting Analysis

Following registration, we created tools to visually and quantitatively analyze metastases and targeting by CREKA-Cy5 and CREKA-Gd. GFP, color anatomy, CREKA-Cy5, and CREKA-Gd image volumes were all registered together. We built a specialized visualization tool that allowed us to link all the volumes together and replicate operations across volumes. This allows us to examine the GFP volume, find a metastasis using multiplanar reformatting, and examine the corresponding location in each of the other image volumes. The color image allows us to determine the anatomical location of the metastasis and often see some image evidence (typically a white spot) of an abnormality. The CREKA-Cy5 volume allows us to see if the agent got to the tumor with very high sensitivity and to determine if there is potentially heterogeneous distribution. In the registered CREKA-Gd volume, we determined if there was a corresponding, visible MR signal. With this visualization tool, each metastasis essentially became its own experiment.

### 3.6. Data and Hardware

Typically, data collected from cryo-imaging are ~120 GB in total for color, Cy5, and GFP volumes. MRI in vivo and ex vivo scans were 13 MB and 32 MB, respectively. We used a Windows 64-bit workstation with 128 GB RAM and a 16-Core CPU for data processing.

## 4. Results

### 4.1. Registration Results

**Figure 2:**
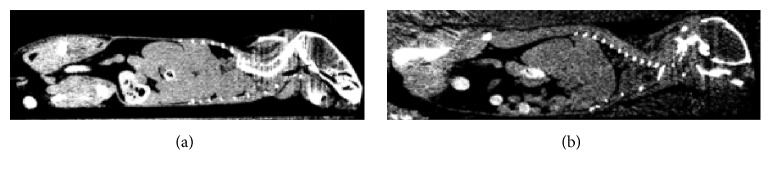
CT assessment of volume change due to freezing. Images are from a postmortem mouse before (a) and after (b) freezing. Images are from similar locations, but one should not compare details in the images. Images are rendered with the same level and window settings, 0 and 400, respectively. Clearly, after freezing, images are darker with reduced CT number in HU indicating volume expansion with freezing.

Since we wanted to understand the deformation process, we examined the result of freezing on CT images, which readily show any changes in tissue density, before and after freezing (Figure 2). We manually aligned the mouse CT volumes before and after freezing with a rigid body transformation to avoid any volumetric or intensity changes, which might affect analyses. After freezing, solid organs are larger (Figure 2(a)) indicating volume expansion. In addition, intensity is reduced indicating a reduction in HU and hence *μ* due to expansion and a decrease in density. We selected similar regions of interest in both volumes, measured mean intensity values, and estimated the volume change using (2). Volume increases for selected organs from the mouse in Figure 2 were liver 8.3 ± 1.5%, left kidney 9.5 ± 2%, right kidney 7.5 ± 2%, and brain 4 ± 1.2%, where standard deviations are obtained from voxelwise measurements of (2). These numbers confirm the need for registration that allows local volume changes.

We optimized registration parameters for our experiments. For rigid and affine registration, we varied parameters consisting of block size, search size, and spacing between cubes and visually inspected results throughout an entire mouse. We found that a block size of 20 × 20 × 20 voxels, a search neighborhood of 30 × 30 × 30 voxels, a block overlap of 3, and 30 iterations worked well. We used a multiscale version of the algorithm with 3 levels, where at each level the block was subsampled by 2 for faster computation and more robust estimation of the rigid and affine registration. Fifty percent of cubes with the highest variance were used to estimate the final global transformation using trimmed least square regression. Algorithm performance was more sensitive to block size than other parameters. Parameters worked very well for all mice with no need to optimize parameters for each mouse.

**Figure 3:**
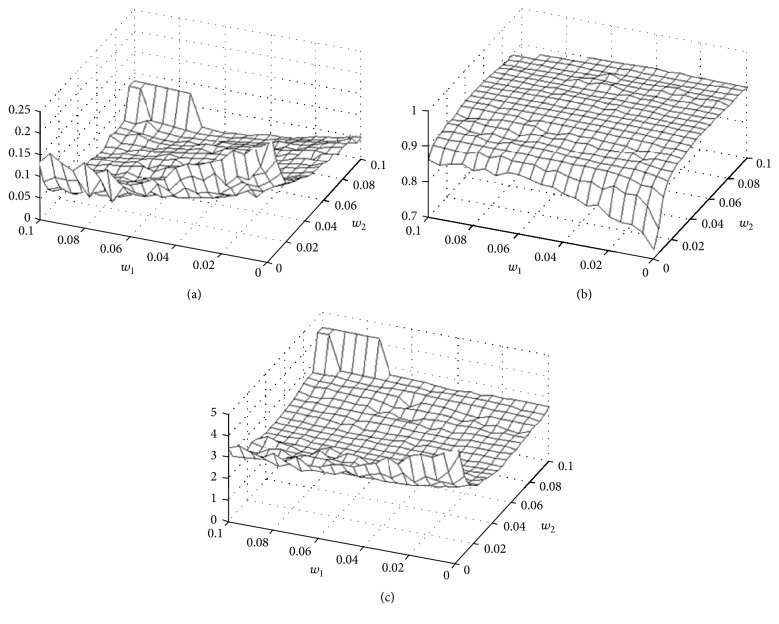
MRI-cryo-quantitative registration quality in lungs. From (a)–(c), plots are volume difference, Dice, and surface distance. We performed 421 registration experiments by varying weights *w*_1_ and *w*_2_ associated with bending energy and Jacobian determinant regularization terms, respectively. Lungs were interactively segmented in cryo- and MRI volumes to enable quantitative assessments for registration quality.

FFD registration was more sensitive to its parameters. Following preliminary experiments, we settled on a grid point spacing of 5 × 5 × 5 voxels, typically giving about 130 × 57 × 48 grid points in a mouse. We used three-levels multiscale approach giving effectively 1/64th the original volume at the starting lowest resolution and stopping criteria of 500 maximum iterations and cost function tolerance of 0.001. Typically, the tolerance was reached before the maximum number of iterations. To investigate the role of the regularization parameters, *w*_1_ and *w*_2_, we did a grid search and quantitatively evaluated registration quality in lungs (Figure 3). We heuristically identified a set of parameters suitable for all mice. Over a broad range of *w*_1_ and *w*_2_ values, registration quality measures were fairly flat. Visual quality was deemed acceptable. Considering all of the objective measures, we chose *w*_1_ = 0.045 and *w*_2_ = 0.055. This gave surface distance error of 0.21 ± 0.16 mm. This corresponds to an error of only about 1.5 voxels in a region most fraught with deformation. Without regularization, metrics were very much degraded. Values of *w*_1_ and *w*_2_ greater than 0.1 resulted in much degraded registrations; hence the figure only shows results in the 0–0.1 range.

**Figure 4:**
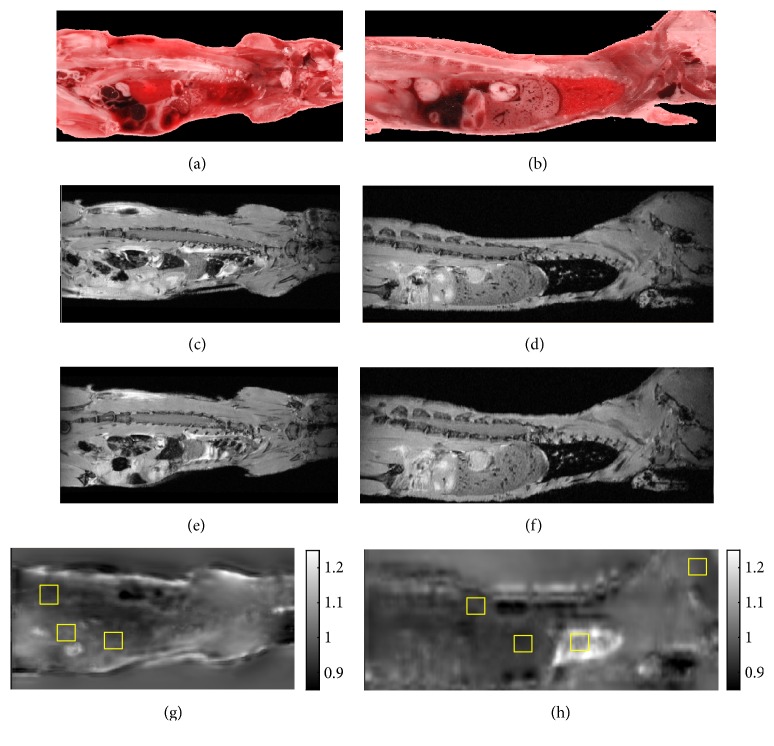
Evaluation of tissue expansion from spatial maps of the determinant of Jacobian. MR images are registered using rigid body (c, d) and affine plus nonrigid (e, f) to the reference color cryo-image volume ((a) coronal, (b) sagittal). The determinant of the Jacobian (g, h) can be interpreted as fractional volume change, with values <1 and >1 representing volume expansion and shrinkage, respectively. The color bar ranges from 0.85 to 1.25. In (g), within regions of interest (ROIs, yellow boxes) from left to right, values are muscle 0.93 ± 0.03, kidney 0.94 ± 0.02, and spleen 0.95 ± 0.02, where standard deviations come from voxelwise analysis in the ROIs. In the *XZ* (h) view, ROIs from left to right are spinal cord 0.91 ± 0.03, liver 0.92 ± 0.01, lungs 1.18 ± 0.05, and brain 0.97 ± 0.01. A value of 0.92 in the liver corresponds to ~8% expansion with freezing. The lung deflated and shrunk. Other animals showed similar expansion/shrinkage trends.

**Figure 5:**
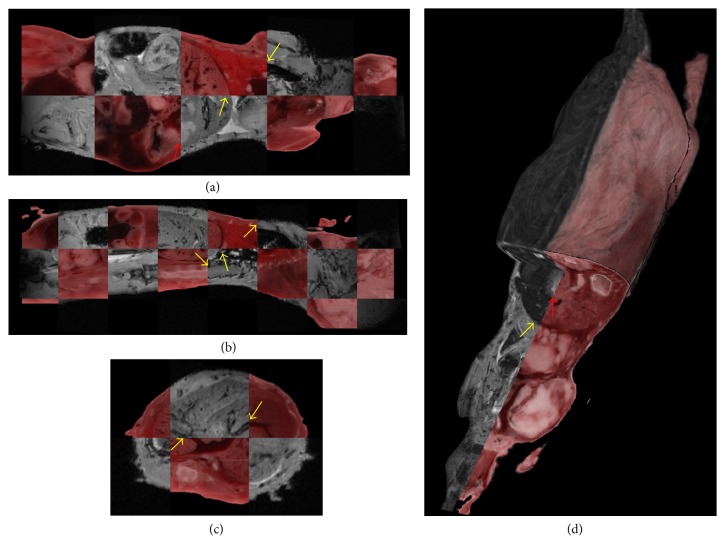
Visual evaluation of registration quality for whole mouse. Figure shows multiplanar checker board view of registration results. In (a) yellow arrows point at edges of lungs and liver in *XY* view; red arrows point at parts of intestine showing misregistration. In (b) arrows point at edges of lungs, liver, and spinal cord in *XZ* view, and in (c) a blood vessel in liver is shown in *YZ* view. (d) 3D visualization to assess registration accuracy in 3D. Yellow arrows point at liver edge. Red arrow points at parts of intestines showing misregistration.

Figures 4 and 5 show that we get good registration quality over the whole mouse using parameters optimized as above for the lung. Registration appears to accommodate the expansion due to freezing. We routinely used the orthogonal checkerboard views (Figure 5 and supplemental Movie 1) for visual assessment. As shown in the figure, good features for assessment include blood vessels and organ edges. Even though parameters were optimized for lungs, other anatomical features throughout the mouse registered well. One can clearly see continuity of lungs, liver, and blood vessels when transitioning from cryo-images to MRI. Other mice gave similar results. Spatial maps of the determinant of the Jacobian from nonrigid registration (Figure 4) suggested expansion with freezing similar to that obtained in the CT experiment.

Quantitative assessments of registration throughout the mouse were also positive. For acceptable *w*_1_ and *w*_2_ weights, registration of the lungs was accurate, giving an average distance between segmented surfaces of ~0.2 mm. In addition, we used metastases as fiducials. Since we did not consider fluorescent images in registration, we consider GFP-labeled metastases to be independent measures of registration accuracy, and arguably the best indicator since they are the ultimate features of interest. We picked 20 tumors from the GFP cryo-volumes from all over the mouse with sizes ranging from 0.2–4 mm in diameter. Corresponding tumors were also visible in MRI. Distances between tumor centers were 0.29 ± 0.21 mm. This is deemed sufficiently accurate to establish unequivocal correspondence of tumors.

**Figure 6:**
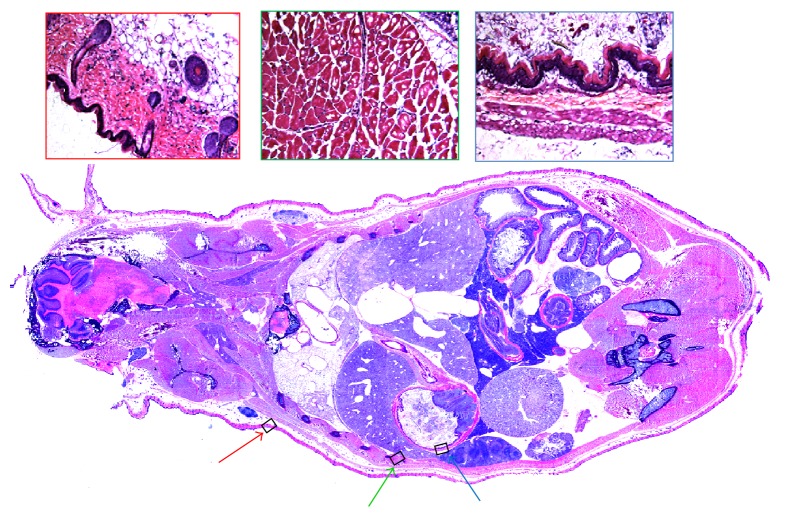
Whole mouse histology section acquired with adhesive film. H&E stained. Figures on the right in red, green, and blue boxes are zoomed images obtained at arrows having the same colors in the whole mouse histology section.

**Figure 7:**
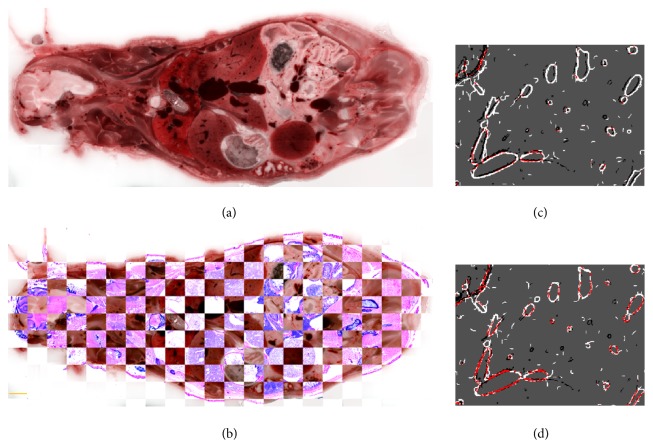
Registration quality of whole mouse histology to color episcopic cryo-image. A whole mouse histological section was obtained with an adhesive film technique and registered to the corresponding color cryo-image (a). A checkerboard display (b) shows that many edges line up in the two registered images. A high resolution checkerboard with 400 *μ*m on a side was used to roughly estimate registration accuracy by manually measuring distances between edges. Images (c) and (d) show significant edges from cryo-images (black) and histology (white) after affine (c) and nonrigid (d) registration. Pixels colored red indicate an overlay of both black and white edges. The amount of red pixels indicates excellent overlap of edges and improvement with nonrigid registration. Bar is 2750 *μ*m.

We acquired histological sections and accurately registered them to the corresponding block face of tissue (Figures 6 and 7). In Figure 6, we show a whole mouse section and high resolution insets. In Figure 7, we evaluate registration accuracy between the cryo-image and histology using checkerboard and edge maps. We identified distances between manually identified corresponding points in cryo- and histology images. Results in microns for (min, max, and average) distances were (44, 110, and 77) and (11, 34, and 22) for rigid and nonrigid registration, respectively, values that compare very favorably to the error associated with selection of features, about ±1 pixel (±11 *μ*m).

### 4.2. Multimodality Imaging of Metastases with Targeted Imaging Agents

**Figure 8:**
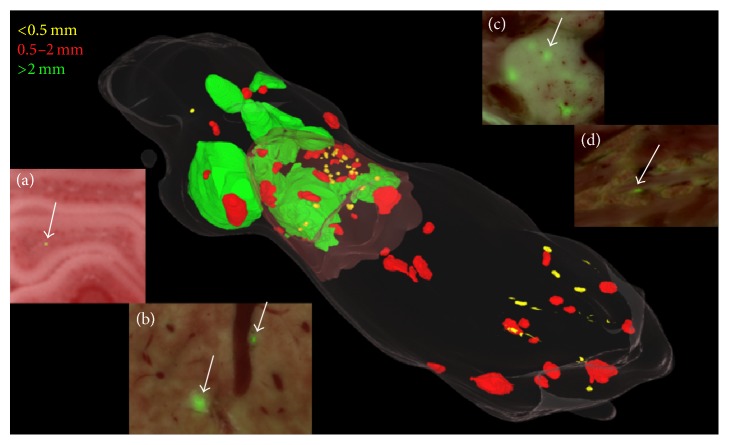
Distribution of metastases in the mouse tumor model. Segmented metastases are shown with volume rendering. Color coding of metastases is based on size as follows: <0.5 mm (yellow), 0.5–2 mm (red), and >2 mm (green). There are 166 metastases in this mouse with 92 yellow, 63 red, and 11 green. Overlaid GFP and episcopic color images show tumor and anatomy. Examples of metastases are in (a) lungs, (b) vertebra, (c) liver, and (d) brain.

We evaluated the distribution and size of metastases in our fat-pad mouse metastases model using the cryo-imaging GFP volume. Tumors were segmented using an interactive tool.  Figure 8 shows the range of sizes and locations of metastases in our mouse model. In the 3D rendering, tumor size is color coded with diameters <0.5 mm (yellow), 0.5–2 mm (red), and >2 mm (green). Insets of 2D views of fused GFP and color episcopic images show the appearance of tumors in selected organs. Across three mice, the average number of metastases was 156 ± 13, ranging in diameter from 0.1 to 6 mm. Across all mice, about 65% of tumors were found in the lung. Tumors were found in organs throughout the mouse with prevalence: lungs > liver ≈ bone marrow > spleen ≫ muscle. Nearly all of the larger tumors were found in lung and liver. Throughout the brain, we found what appears to be a single tumor cell (inset).

**Figure 9:**
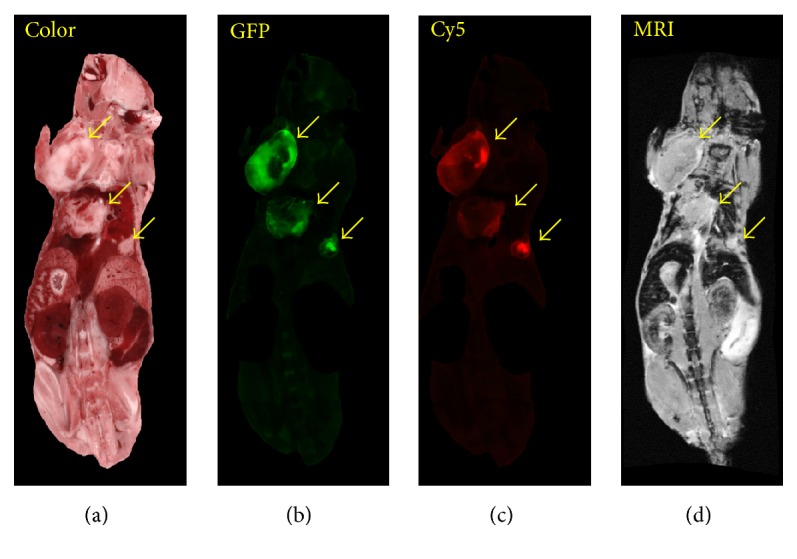
Multimodality analysis of large metastases targeting. From left to right are color episcopic anatomy, green fluorescence GFP tumor, red fluorescence CREKA-Cy5, and MRI CREKA-Gd. Large tumors (arrows) visible in bright field and GFP images are labeled in Cy5 and MRI images. In these images, we masked out the GI tract and skin, which tend to have a lot of auto fluorescence. The imaging agents tend to label the edges of bigger metastases and all of smaller ones.

**Figure 10:**
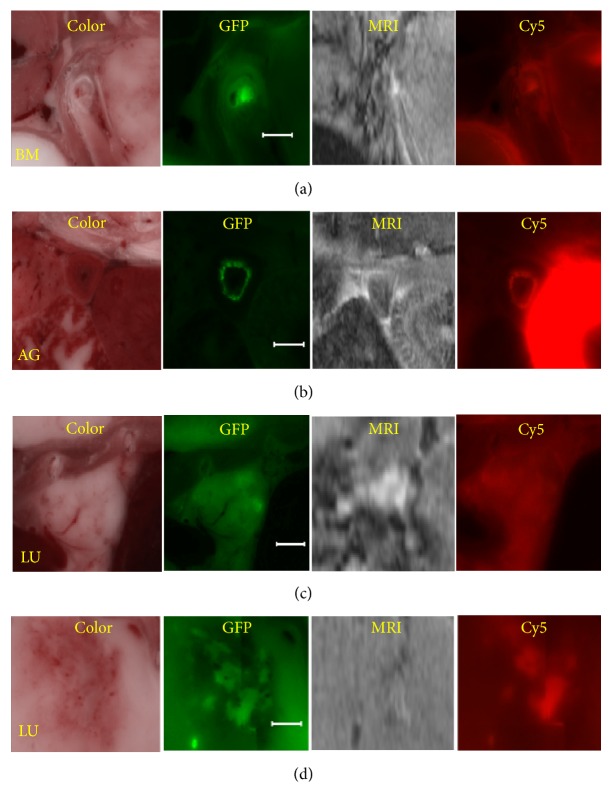
Multimodality analysis of micrometastases targeting. Examples of micrometastases were found in bone marrow (BM), adrenal glands (AG), and lungs (LU). In (a, b, c), CREKA-Cy5 and CREKA-Gd successfully labeled these micrometastases. The super bright region in the AG image is adjoining kidney which tends to accumulate CREKA-Cy5. In (d), an example where CREKA-Cy5 labeled metastases but not CREKA-Gd. Bar is 1 mm.

Following registration and tumor segmentation, we used interactive visualization tools to examine peptide targeting (Figures 9 and 10 and supplemental Movie 2). Using the software, one can identify a metastasis in episcopic color and fluorescence GFP images, examine the corresponding location in registered, linked red fluorescence and MRI volumes, and determine the presence of CREKA-Cy5 and MRI CREKA-Gd signals.  Figure 9 shows example images from all 4 registered volumes. Arrows point to 3 large metastases that are clearly labeled by CREKA-Cy5 and CREKA-Gd. As seen in the figure, peptide tends to label the edges of larger metastases. Interestingly, regions of high imaging agent signals tend to overlap with those of high GFP signals. Smaller metastases are more uniformly labeled. With the interactive visualization tool, we can zoom to a small GFP tumor and inspect for CREKA-Cy5 and GREKA-Gd signal.  Figure 10 includes multiple examples of 0.2–2 mm metastases. Figures 10(a)–10(c) show instances where metastases were labeled with both CREKA-Cy5 and CREKA-Gd. Correspondence between GFP and Cy5 is remarkable with identical shapes. Please note that the very bright region in the Cy5 image of the adrenal gland (Figure 10(b)) corresponds to uptake in the kidney. This metastasis has a very bright enhancing rim in GFP, CREKA-Cy5, and CREKA-Gd, indicative of nonhomogeneous tissue. This was typical for adrenal gland tumors but very atypical for small tumors in other organs where enhancement was more uniform. Many micrometastases were also detected by both CREKA-Gd and CREKA-Cy5 in the lungs, and a 2 mm example is shown (Figure 10(c)). In a mouse studied in great detail, we determined that in the MR images every tumor in the lung >0.3 mm^2^ had visible signal and that some metastases as small as 0.1 mm^2^ were also visible. In general, CREKA-Cy5 detected more metastases than CREKA-Gd (Figure 10(d)). Here we see GFP tumor and CREKA-Cy5 labeling but no evidence of CREKA-Gd signal. Some very small GFP micrometastases (~10–15% per mouse) smaller than 0.1 mm^2^ were not detectable in both CREKA-Cy5 and MRI.

**Figure 11:**
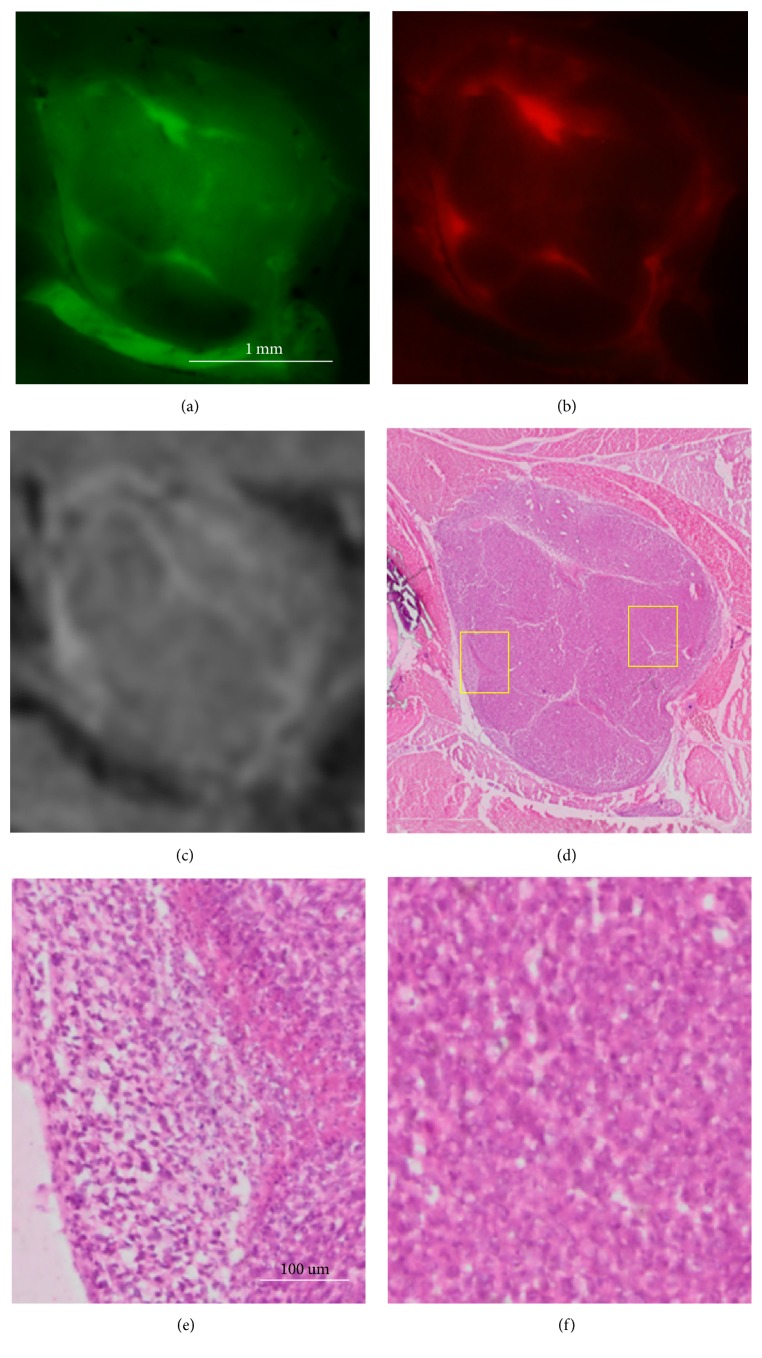
cryo-imaging and histology of a tumor with inhomogeneous GFP distribution and CREKA-Cy5 and CREKA-Gd labeling. The tumor consisting of green fluorescence GFP, red fluorescence CREKA-Cy5, MRI CREKA-Gd, and histology H&E is shown in (a–d), respectively. Magnified histology images in (e) and (f) are from the left and right regions identified in (d) with high and low labeling, respectively. (e) contains tumor cells and a blood vessel, presumably leading to hyperintense labeling. (f) shows a region of low labeling signal intensity comprised of noncancerous tissue. Scale bar = 100 *μ*m.

Our histology registration enables further determination of tumor heterogeneity. In Figure 11, we show a single large tumor interrogated with histology as well as other imaging modalities. The GFP tumor (a) is brightly labeled in both MRI CREKA-Gd (c) and fluorescence CREKA-Cy5 (b) images. In all three images of this heterogeneous tumor, there is remarkable correlation between GFP and Cy5 signals and good correlation with the Gd signal in MRI. The registered H&E histology image (d) exactly conforms to the green and red fluorescence cryo-images. In (e), histology shows cancer cells in the periphery. In (f), the center of the tumor corresponds to the nonenhancing region in the GFP, CREKA-Cy5, and MRI CREKA-Gd images. This region shows less tumor cells with no evidence of blood vessels. In addition, from zoomed in images, one can see that there are immune cells at the edge of the tumor indicating inflammation, which supports that 4T1 induces inflammation in mice models.

## 5. Discussion

This unique demonstration is made possible with our multimodality imaging platform, CITP, built on cryo-imaging. The unique feature of our software platform is that it enables at a whole-body level and with high resolution the localization of micrometastases in the body by employing single cell resolution cryo-imaging as a key modality and provides tools for studying the targeting efficiency at each micrometastasis. To our knowledge, such a platform has never been reported before. Although our example was molecular MRI, it is feasible extending the approach to other modalities, for example, FMT, CT, and PET. Due to resolution limitations, there will be challenges associated with registration of these other modalities to cryo-images. Our platform acts as the perfect bridge between in vivo modalities such as MRI, lower-resolution PET, and very high resolution histology which is impractical on a whole mouse.

The platform is especially well suited for evaluation of imaging agents targeting metastatic tumors. Metastatic cancer is particularly difficult to analyze, as tumors can be small, scattered over large regions of tissue, making it impossible to obtain a gold standard assessment with histology. cryo-imaging (CryoViz, BioInVision) provides a unique opportunity to image vast volumes, as large as an entire mouse, with single tumor cell sensitivity. In this study, we used the 4T1-GFP-Luc2 cell line, which allows one to image developing tumor with bioluminescence over time, and GFP labeled metastases with cryo-imaging. Cryo-imaging provided episcopic color, GFP tumor, and red fluorescence targeted imaging agent (CREKA-Cy5). Of course, all three cryo-image data sets were exactly registered, as they are obtained at the same time. In addition, selected histology sections were obtained with the adhesive film method, which preserved spatial integrity, allowing registration with high accuracy to corresponding cryo-images. Finally, we evaluated molecular MRI with a targeted imaging agent, CREKA-Gd. All told that we had 5 types of image data (color anatomy, green fluorescent tumor, red fluorescent CREKA-Cy5, MRI CREKA-Gd, and histology); all aimed at understanding image agent targeting of metastatic cancer.

Our methods have successfully met the challenges of image registration between MRI and cryo-imaging. We have found that nonrigid registration is required. As shown in Figures 4 and 5, there is expansion and relocation of tissues due to freezing. The air-filled lungs compress and create the most challenging region for registration. We detected displacements of the kidneys, spleen, and intestine. As has been reported by others [14], we found that correlation block matching for affine registration was fast and robust as compared to mutual-information-based affine registration. This algorithm uses absolute cross correlation to locally match edges inside each block. Since edge features in MRI are also seen in cryo-color images, this method works well for our needs. In our experience, we found that getting close with an affine transform is a prerequisite for good nonrigid registration. Nonrigid, mutual information, B-spline registration, does a good job registering cryo-images to MRI from both qualitative (Figure 5) and quantitative analyses (Figure 3). Proper tuning of *w*_1_ and *w*_2_ values was required, especially to get good local registration in the lungs and liver where most metastases were present. Registration accuracy in the stomach, spleen, some bones, and parts of the kidneys was sometimes worse, probably due to relative shifts between organs due to freezing. Nevertheless, misregistration was not a big problem as errors on the order of 0.3 mm were insufficient to cause ambiguity when matching tumors between cryo- and MRI volumes. Specialized experimental and nonrigid registration methods allowed us to very accurately register (within about 22 *μ*m) whole mouse histological sections to cryo-images [25].

The CITP software platform including registration, visualization, and quantitative analysis enabled unique analyses of image agent targeting. With registered data, we could interactively visually interrogate the GFP volume for tumor; zoom to 2D images at high resolution; determine if CREKA-Cy5 was present in the red fluorescence image with very high sensitivity; and then determine if there was signal present in the MRI CREKA-Gd image. Supplemental Movie 2 illustrates the visual interaction. Optionally, we could also examine histology of the same tumor registered exactly to the cryo-data. Essentially, each metastatic tumor became a labeling experiment onto itself. CITP will allow us to identify true-positive and false-negative molecular imaging of tumors. In theory, it should also allow us to identify false positive detection.

In these preliminary experiments, CREKA-Cy5 and CREKA-Gd showed remarkable ability to label metastases.  Figure 10 included examples of metastases detected by fluorescence imaging with CREKA-Cy5 that were not highlighted in MRI CREKA-Gd. It is likely that this is due to enhanced sensitivity of fluorescence cryo-imaging relative to MRI. However, there might be less CREKA-Gd accumulation, as it is a larger molecule than CREKA-Cy5.

Detection of micrometastases depends on multiple factors, including the number of targeted molecules needed to generate sufficient contrast and the sensitivity of the imaging modality. Since CREKA targets fibrin-fibronectin complexes in blood clots in the tumor microenvironment, detectability of the tumor depends on the number of these complexes in the tumor microenvironment. For some very small GFP micrometastases (~10–15% per mouse) that were not detectable in both red fluorescence and MRI, we did not find visible blood vessels nearby which are often seen in other tumors. An attractive hypothesis is that these tumors had insufficient blood flow to form clots continuing fibrin-fibronectin complexes.

Labeling of tumor is often heterogeneous. Fluorescence CREKA-Cy5 and MRI CREKA-Gd images oftentimes show very similar heterogeneous features (Figures 7, 8, and 11). Interestingly, both tend to label GFP hot zones, indicating that there is more labeling where there is an abundance of active tumor cells. Heterogeneity warrants additional study and cryo-imaging plus histology offers an excellent opportunity. Occasionally, we found large tumors that were clearly visible in episcopic color and CREKA-Cy5 but not GFP. Such tumors either lost the ability to produce GFP in the mouse or were not labeled when they were injected.

There did not appear to be untoward effects from the long scan time, high resolution ex vivo MRI scan. We checked for potential degradation of the GFP signal using a whole mouse, Maestro™ multispectral fluorescence system. Intensity of GFP was unchanged over 2 hr postmortem. Although MR relaxivities are known to change between in vivo and ex vivo, particularly as a function of temperature [26], we did not detect important changes in Gd contrast relative to background tissue. Possibly, there could be degradation of histological sections over this time. However, we observed good cellular detail. There is a potential advantage. After 2 hr postmortem, some tissues stiffened due to rigor mortis [27], possibly reducing deformation during freezing.

We have identified some limitations that can be addressed with experimental and software approaches. First, image registration between MRI and cryo-images is good but imperfect. This does not hamper visual interpretation as one can normally identify unambiguously tumor matches following registration. It does impair the ability to perform automatically an analysis of image intensities. It will be interesting to apply new “sliding organ” registration methods [28–31], which could give improved results. Another approach could be to perfuse the mice with cryo-protectant prior to freezing, as sometimes done in autoradiography. Cryo-protectant will reduce ice crystal formation and lead to verification with less tissue volume expansion [32, 33]. Filling the lungs with freezing medium could limit deformation. Yet another approach would be to image excised organs eliminating sliding and possibly reducing deformation. Second, we segmented GFP tumors manually, a very laborious process. The 4T1-GFP-Luc2 cell line was not very bright in GFP. Other tumor cell lines using a single EGFP gene reporter without luciferase are sometimes 10–15 times as bright. Such cells would be above auto fluorescence making automatic segmentation with advanced image analysis techniques possible. This would greatly enhance pipeline throughput. Third, it will also be possible to improve specificity of MR imaging by enhancing the contrast of the Gd signal relative to normal tissue. Methods include using a lower magnetic field strength, alternative acquisition schemes, and MR finger printing developed at our institution [34], which as currently applied has an advantage that it can capture all T1 and T2 information in a single time-efficient acquisition allowing one to create a signature for Gd, possibly with improved differences to background. If we use a single T1-weighted acquisition, the differences between Gd and background could be diminished. Fourth, another enhancement will be the introduction of immunohistochemistry. This will provide information about tumor cell differentiation, inflammation, and necrosis along with the presence of targeted molecules providing further information about tumor microenvironment and the targeted molecules. Fifth, as described above, occasionally tumors were not labeled with GFP. As it is likely that these arose from cells not labeled with GFP, this phenomenon should be reduced by careful FACS sorting of tumor cells prior to implantation.

## 6. Conclusion

Initial results with the multimodality CITP are very promising. We have shown that the platform uniquely characterizes targeting of imaging agents to metastatic tumors. Because of its unique ability to image and quantify small metastases, we believe that the platform will be uniquely suited for evaluation and optimization of pipelines of technologies (imaging agents, imaging methods, theranostics, therapeutics, tumor models, etc.) important for detecting, understanding, and treating metastatic cancer.

## Figures and Tables

**Algorithm 1:**
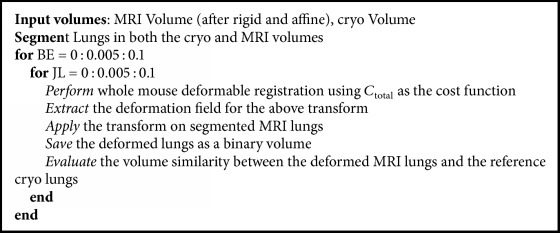
Pseudocode to find optimal parameters for cost function using lungs.

## Supplementary Materials

Supplementary Materials Movie 1: registration quality is shown by displaying side-by-side registered MRI and color cryo-image volumes (mp4, 1.61 MB). Movie 2: interactive analysis platform. GFP-labeled metastatic tumors are segmented in the green fluorescence cryo-image volume and color coded as to size. One can examine a tumor of interest and go to the registered MRI volume to examine the corresponding MR signal. The zoomed tumor has a diameter of ~4 mm. CREKA-Gd can be seen at the edges of the tumor (MP4, 11.5 MB).

## References

[1] Zhou Z., Qutaish M., Han Z. (2015). MRI detection of breast cancer micrometastases with a fibronectin-targeting contrast agent. *Nature Communications*.

[2] Wuttisarnwattana P., Gargesha M., Van'T Hof W., Cooke K. R., Wilson D. L. (2016). Automatic stem cell detection in microscopic whole mouse cryo-imaging. *IEEE Transactions on Medical Imaging*.

[3] Roy D., Steyer G. J., Gargesha M., Stone M. E., Wilson D. L. (2009). 3D cryo-imaging: A very high-resolution view of the whole mouse. *Anatomical Record*.

[4] Burden-Gulley S. M., Qutaish M. Q., Sullivant K. E. (2011). Novel cryo-imaging of the glioma tumor microenvironment reveals migration and dispersal pathways in vivid three-dimensional detail. *Cancer Research*.

[5] Yang M., Baranov E., Jiang P. (2000). Whole-body optical imaging of green fluorescent protein-expressing tumors and metastases. *Proceedings of the National Acadamy of Sciences of the United States of America*.

[6] Oyajobi B. O., Muñoz S., Kakonen R. (2007). Detection of myeloma in skeleton of mice by whole-body optical fluorescence imaging. *Molecular Cancer Therapeutics*.

[7] Kaijzel E. L., Van Der Pluijm G., Löwik C. W. G. M. (2007). Whole-body optical imaging in animal models to assess cancer development and progression. *Clinical Cancer Research*.

[8] Hoffman R. M. (2002). In vivo imaging of metastatic cancer with fluorescent proteins. *Cell Death & Differentiation*.

[9] Ishii T., Ishii M. (2011). Intravital two-photon imaging: a versatile tool for dissecting the immune system. *Annals of the Rheumatic Diseases*.

[10] Alexander S., Weigelin B., Winkler F., Friedl P. (2013). Preclinical intravital microscopy of the tumour-stroma interface: Invasion, metastasis, and therapy response. *Current Opinion in Cell Biology*.

[11] Conway J. R. W., Carragher N. O., Timpson P. (2014). Developments in preclinical cancer imaging: Innovating the discovery of therapeutics. *Nature Reviews Cancer*.

[12] Kawamoto T. (2003). Use of a new adhesive film for the preparation of multi-purpose fresh-frozen sections from hard tissues, whole-animals, insects and plants. *Archives of Histology and Cytology*.

[13] Duchon C. E. (1979). Lanczos filtering in one and two dimensions. *Journal of Applied Meteorology*.

[14] Ourselin S., Roche A., Subsol G., Pennec X., Ayache N. (2001). Reconstructing a 3D structure from serial histological sections. *Image and Vision Computing*.

[15] Ourselin S., Stefanescu R., Pennec X., Dohi T., Kikinis R. (2002). Robust registration of multi-modal images: towards real-time clinical applications. *Medical Image Computing and Computer-Assisted Intervention — MICCAI 2002*.

[16] Rueckert D., Sonoda L. I., Hayes C., Hill D. L. G., Leach M. O., Hawkes D. J. (1999). Nonrigid registration using free-form deformations: application to breast MR images. *IEEE Transactions on Medical Imaging*.

[17] Pluim J. P. W., Maintz J. B. A. A., Viergever M. A. (2003). Mutual-information-based registration of medical images: a survey. *IEEE Transactions on Medical Imaging*.

[18] Studholme C., Hill D. L. G., Hawkes D. J. (1999). An overlap invariant entropy measure of 3D medical image alignment. *Pattern Recognition*.

[19] Wahba G. Spline models for observational data.

[20] Rohlfing T., Maurer C. R., Bluemke D. A., Jacobs M. A. (2003). Volume-preserving nonrigid registration of MR breast images using free-form deformation with an incompressibility constraint. *IEEE Transactions on Medical Imaging*.

[21] Cachier P., Rey D., Delp S., DiGoia A., Jaramaz B. (2000). Symmetrization of the non-rigid registration problem using inversion-invariant energies: application to multiple sclerosis. *Medical Image Computing and Computer-Assisted Intervention—MICCAI 2000*.

[22] Akbarzadeh A., Gutierrez D., Baskin A. (2013). Evaluation of whole-body mr to ct deformable image registration. *Journal of Applied Clinical Medical Physics*.

[23] Dice L. R. (1945). Measures of the amount of ecologic association between species. *Ecology*.

[24] Klein A., Andersson J., Ardekani B. A. (2009). Evaluation of 14 nonlinear deformation algorithms applied to human brain MRI registration. *NeuroImage*.

[25] Burden-Gulley S. M., Qutaish M. Q., Sullivant K. E. (2013). Single cell molecular recognition of migrating and invading tumor cells using a targeted fluorescent probe to receptor PTPmu. *International Journal of Cancer*.

[26] Bjrnerud A., Johansson L. O., Briley-Sb K., Ahlstrm H. K. (2002). Assessment of T1 and T2∗ effects in vivo and ex vivo using iron oxide nanoparticles in steady state - Dependence on blood volume and water exchange. *Magnetic Resonance in Medicine*.

[27] Su R., Ermilov S. A., Liopo A. V., Oraevsky A. A. Optoacoustic 3D visualization of changes in physiological properties of mouse tissues from live to postmortem.

[28] Pace D. F., Enquobahrie A., Yang H., Aylward S. R., Niethammer M. Deformable image registration of sliding organs using anisotropic diffusive regularization.

[29] Risser L., Vialard F.-X., Baluwala H. Y., Schnabel J. A. (2013). Piecewise-diffeomorphic image registration: Application to the motion estimation between 3D CT lung images with sliding conditions. *Medical Image Analysis*.

[30] Pace D. F., Aylward S. R., Niethammer M. (2013). A locally adaptive regularization based on anisotropic diffusion for deformable image registration of sliding organs. *IEEE Transactions on Medical Imaging*.

[31] Liu Y., Zhou B., Qutaish M., Wilson D. L. Microscopic validation of whole mouse micro-metastatic tumor imaging agents using cryo-imaging and sliding organ image registration.

[32] Zhmakin A. I. (2008). Physical aspects of cryobiology. *Physics-Uspekhi*.

[33] Rabin Y., Wolmark N., Taylor M. J. (1998). Thermal expansion measurements of frozen biological tissues at cryogenic temperatures. *Journal of Biomechanical Engineering*.

[34] Ma D., Gulani V., Seiberlich N. (2013). Magnetic resonance fingerprinting. *Nature*.

