# Supplementary material for: Cryo-Imaging and Software Platform for Analysis of Molecular MR Imaging of Micrometastases
Source: Int J Biomed Imaging. 2018 Apr 1;2018:9780349. doi: 10.1155/2018/9780349 (PMC5899875; doi:10.1155/2018/9780349)
Supplement: Supplementary Materials — Movie 1: registration quality is shown by displaying side-by-side registered MRI and color cryo-image volumes (mp4, 1.61 MB). Movie 2: interactive analysis platform. GFP-labeled metastatic tumors are segmented in the green fluorescence cryo-image volume and color coded as to size. One can examine a tumor of interest and go to the registered MRI volume to examine the corresponding MR signal. The zoomed tumor has a diameter of ~4 mm. CREKA-Gd can be seen at the edges of the tumor (MP4, 11.5 MB). [file 9780349.f1.zip › 9780349.f1.docx]

**Movie 1.** Registration quality is shown by displaying side-by-side registered MRI and color cryo image volumes.

(mp4, 1.61 MB).

**Movie 2.** Interactive analysis platform. GFP-labeled metastatic tumors are segmented in the green fluorescence

cryo-image volume and color coded as to size. One can examine a tumor of interest and go to the registered MRI

volume to examine the corresponding MR signal. The zoomed tumor has a diameter of $\sim$4 mm. CREKA-Gd can be

seen at the edges of the tumor. (MP4, 11.5 MB).
